# Molecular insights into the regulation of iron metabolism during the prenatal and early postnatal periods

**DOI:** 10.1007/s00018-012-1018-1

**Published:** 2012-05-13

**Authors:** Paweł Lipiński, Agnieszka Styś, Rafał R. Starzyński

**Affiliations:** Department of Molecular Biology, Institute of Genetics and Animal Breeding, Polish Academy of Sciences, Jastrzębiec, ul. Postępu 1, 05-552 Magdalenka, Poland

**Keywords:** Iron, Fetus, Placenta, Early postnatal development, Hepcidin

## Abstract

Molecular iron metabolism and its regulation are least well understood in the fetal and early postnatal periods of mammalian ontogenic development. The scope of this review is to summarize recent progress in uncovering the molecular mechanisms of fetal iron homeostasis, introduce the molecules involved in iron transfer across the placenta, and briefly explain the role of iron transporters in the absorption of this microelement during early postnatal life. These issues are discussed and parallels are drawn with the relatively well-established system for elemental and heme iron regulation in adult mammals. We conclude that detailed investigations into the regulatory mechanisms of iron metabolism at early stages of development are required in order to optimize strategies to prevent neonatal iron deficiency. We propose that newborn piglets represent a suitable animal model for studies on iron deficiency anemia in neonates.

## An outline of systemic and cellular iron homeostasis in adults

### Systemic iron homeostasis: hepcidin–ferroportin axis

**Fig. 1:**
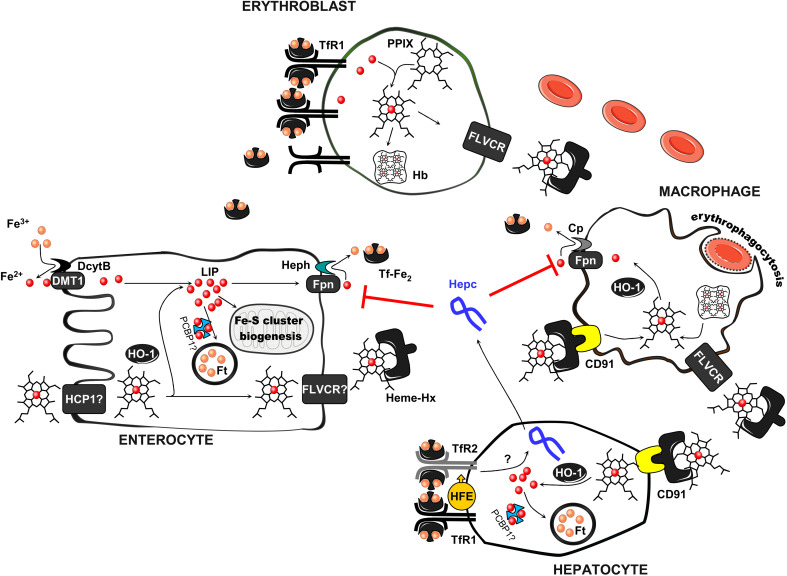
Iron homeostasis in absorptive enterocytes, macrophages, erythroblasts, and hepatocytes and its routes of circulation in the organism. Non-heme iron absorption occurs in intestinal epithelial cells (enterocytes) in the duodenum. The first step in the transport of iron across the apical membrane of enterocytes is ferric (Fe^3+^) to ferrous (Fe^2+^) iron reduction, catalyzed by the membrane-associated ferrireductase DcytB. Ferrous iron is subsequently transported into the enterocyte via the divalent metal transporter 1 (DMT1)-dependent pathway. Heme, another source of dietary iron, is also taken up by enterocytes, although its receptor/transporter has not been fully characterized. A proton-coupled folate transporter/heme carrier protein 1 (PCFT/HCP1) has been proposed as being primarily responsible for heme uptake, but recent data show that it mainly serves as a folate transporter and has a lower affinity for heme. After uptake, heme is catabolized by inducible heme oxygenase 1 (HO-1)—to iron, biliverdin, and carbon monoxide. The released iron is subsequently used for cellular needs (e.g., for iron–sulfur cluster biogenesis in mitochondria), stored inside the cell in ferritin (which probably requires the chaperone PCBP1 (poly (rC) binding protein 1) to delivers iron to Ft), or exported into the circulation by the iron exporter ferroportin (Fpn). Iron export from enterocytes also requires hephaestin (Heph), a multicopper oxidase, which oxidizes Fe^2+^ to Fe^3+^, prior to iron binding by transferrin in the blood (Tf). Iron bound to transferrin is taken up by most cells via receptor-mediated endocytosis. There are two known transferrin receptors (TfRs): TfR1, which is present in all cell types, and TfR2 mostly expressed in hepatocytes. To prevent heme toxicity and cell death, hematopoietic and most non-hematopoietic cells express feline leukemia virus subgroup C cellular receptor (FLVCR1), which mediates heme export. This is of particular importance in the removal of heme from erythroid progenitor cells that have a high iron requirement (e.g., by increased TfR1 expression) for hemoglobin (Hb) production. Heme present in the blood circulation is cleared by hemopexin (Hp). The heme–hemopexin complex is taken up by hepatocytes and macrophages of the reticuloendothelial system via CD91-mediated endocytosis. Since there is no natural pathway to excrete excess iron from the organism and iron uptake is limited, the recycling of iron from heme released from red blood cells after erythrophagocytosis is the main source of the element to fulfil daily requirements. Iron trafficking is controlled by the key iron regulatory hormone hepcidin. Its expression, which takes place mainly in hepatocytes, is precisely regulated and depends, e.g., on transferrin saturation. Hepcidin can bind to Fpn, causing its internalization and degradation, hence decreasing iron export from enterocytes and other cell types into the plasma

Iron is an essential element for biological processes since it participates in multiple enzymatic reactions as a part of iron–sulfur clusters, heme prosthetic groups, and other iron-containing centers, which makes it indispensable for almost all living organisms. However, iron–oxygen interaction, which is a source of free radicals generated by the Fenton reaction, makes iron a doubled-edged sword in an oxygen environment. Furthermore, since there is no natural pathway for excreting excess iron from the organism, systemic iron homeostasis must be very tightly controlled in order to ensure coordinated iron absorption by enterocytes, reutilization in macrophages of the reticuloendothelial system, and correct iron redistribution to its site of utilization (mainly for erythropoiesis) or storage (in hepatocytes) [1] (Fig. 1). Iron absorption can be precisely adjusted to the needs of the individual, i.e. enhanced when erythropoiesis is increased or in pregnancy, or suppressed in conditions of iron overload. The key molecule in this regulation is hepcidin. It is synthesized mainly in hepatocytes [2] as a prepropeptide of 84 amino acids. This is subsequently cleaved to 60 aa prohepcidin, which is further processed by furin protease to generate three forms of hepcidin peptide (20 aa, 22 aa, 25 aa), the largest of which is biologically active. After processing to produce the active form, hepcidin is secreted into the circulation. Recent studies have shown that hepcidin can also be locally expressed in the heart, kidney, pancreas, brain, adipose tissue, and pathogen-activated neutrophils and macrophages [3–7].

The likely importance of hepcidin in iron homeostasis was first noted by Pigeon et al*.* [8], who observed that levels of hepcidin mRNA are enhanced in murine hepatocytes in response to iron and after lipopolysaccharide treatment. The key role of hepcidin in the regulation of systemic iron homeostasis was revealed by Nicolas et al*.* [9], who accidentally disturbed the expression of hepcidin by knocking-out its adjacent gene, *Usf2*. Their knockout mice displayed severe, progressive iron overload that resembles the phenotype of HFE knockout mice, a murine model of hereditary hemochromatosis. As expected, the overexpression of hepcidin in transgenic mice resulted in the opposite phenotype, namely, iron deficiency [10]. Subsequently, Roetto et al*.*, working with human patients, identified two mutations in the hepcidin gene, which either led to the production of inactive hepcidin or blocked maturation of the mutated propeptide. Affected individuals suffer from a juvenile hereditary hemochromatosis, which confirms that hepcidin is also an important molecule in human iron homeostasis [11].

Hepcidin acts as a negative regulator of iron absorption or reutilization by binding to ferroportin (Fpn), the only known iron exporter, causing its internalization and degradation, hence decreasing the export of iron from enterocytes and other cell types into the plasma [12, 13]. It was proposed that the binding of hepcidin to ferroportin is dependent on the cysteine residue at position 326 of Fpn, within the extracellular loop [13]. Hepc–Fpn binding activates Janus Kinase2 (Jak2) that in turn phosphorylates Fpn [14], leading to its internalization in clathrin-coated pits, subsequent dephosphorylation, ubiquitination, and finally degradation in lysosomes [15]. Auriac et al*.* [16] challenged this proposal by showing that Fpn internalization is not mediated via clathrin-dependent endocytosis in murine bone marrow-derived macrophages and J774 cells, but occurs via lipid raft-dependent endocytosis. The necessity of Jak2 kinase for hepcidin-induced ferroportin internalization has also been questioned [17]. Furthermore, the tyrosine residues of Fpn that are phosphorylated in hepcidin-mediated Fpn internalization [15] were recently shown not to be necessary for this process in cell cultures [17, 18] or in the mouse model [19]. Various cell types respond differently to hepcidin challenge: macrophages respond more acutely than duodenal enterocytes, in agreement with their central role in iron reutilization and the maintenance of systemic iron homeostasis [20]. Evidence that the hepcidin–ferroportin interaction might not be as simple as was initially thought continues to mount. First, Fpn can also be regulated at the transcriptional and even post-transcriptional level (by the IRP/IRE system) in response to iron fluctuations. Secondly, hepcidin expression is also regulated in response to multiple signals, including systemic iron availability, erythropoiesis, hypoxia, and inflammation. Moreover, new factors that are involved in hepcidin expression, including proteins found to be mutated in various types of hemochromatosis (HFE, HJV, TfR2) or anemia (TMPRSS6), and transcription factors (SMAD4, STAT3), emerge each year. These factors are beyond the scope of this article, but interested readers can refer to a number of excellent reviews [21–23].

### Intracellular iron homeostasis: IRP/IRE regulation

In parallel with the regulation of organismal iron homeostasis via hepcidin, a two-component system exists that acts to maintain cellular iron availability while preventing its toxicity. In mammalian cells, this system is composed of two iron regulatory proteins (IRP1 and IRP2), which post-transcriptionally regulate the expression of iron-related genes by binding to specific sequences called iron responsive elements (IREs) located within the untranslated regions (UTRs) of target mRNAs. Either of the two IRPs can inhibit translation when bound to the single 5′ UTR IRE in the mRNAs encoding iron export (ferroportin—Fpn) and storage (ferritin—Ft) proteins, or they can prevent mRNA degradation when bound to the multiple IREs within the 3′UTR of the mRNA encoding the transferrin receptor 1 (TfR1), an iron uptake molecule. Thus, the binding of the IRPs ensures the coordinated regulation of iron import, export, and storage inside the cell [24]. IREs continue to be found in mRNAs encoding proteins related to iron metabolism, such as erythroid aminolevulinic acid synthase (eALAS or ALAS2) [25], the first and rate-limiting enzyme in the heme synthesis pathway. Within the last decade, single IRE sequences have also been identified in the 3′UTRs of mRNAs encoding myotonic dystrophy kinase-related Cdc42-binding kinase α (MRCKα) [26] and human cell division cycle 14A protein (CDC14A) [27], and the 5′UTRs of the Alzheimer’s amyloid precursor protein [28] and the oxygen-sensing transcription factor Epas1 (Hif2α) [29]. This regulatory network continues to grow and recently 35 novel mRNAs were proposed to be under the control of the IRP/IRE system [30].

The IRE-binding activity of both IRPs responds to cellular iron levels, albeit via distinct mechanisms. IRP1 is a bifunctional protein, which mostly exists in its non IRE-binding, [4Fe–4S] aconitase form that can be regulated by post-translational removal of the Fe–S cluster or its incorporation into a de novo synthesized protein. In contrast, IRP2 is unable to ligate an Fe–S cluster, and its IRE-binding activity is determined by the rate of its proteasomal degradation.

The importance of the IRPs in cellular iron homeostasis is demonstrated by their presence in a wide variety of organisms, including bacteria [31], plants [32], invertebrates, and vertebrates, and also the high sequence conservation of these proteins (64 % amino acid identity in plants and invertebrates and >90 % among mammals) [33]. IRPs are thought to have originated from aconitase, which gained IRE-binding activity by evolution. In contrast to lower eukaryotes, whose genomes do not contain any functional IREs and the aconitase has little or no IRE-binding activity, *Manduca sexta* and *Drosophila melanogaster* were found to have IRE-binding proteins that regulate the expression of ferritin and succinate dehydrogenase subunit B, respectively [34, 35].

In mammalian cells, IRP2 is thought to play a dominant role in the regulation of basal cellular iron homeostasis, since only *Irp2*, but not *Irp1* knockout mice misregulate iron metabolism and display microcytic anemia [36, 37] and neurodegeneration [38]. Interestingly, mice homozygous for a targeted deletion of *Irp2* and heterozygous for a targeted deletion of the *Irp1* gene (*Irp1* ^+*/*−^ *Irp2* ^−*/*−^) develop a much more severe form of neurodegeneration, characterized by axonopathy and subtle vacuolization in several brain areas, particularly in the substantia nigra [39]. Double knockout embryos do not survive gestation, probably because of the abnormal iron sequestration inside the ferritin and decreased iron import via TfR1, and thus functional iron deficiency [40]. Both IRPs are vitally important for ensuring the iron supply to the mitochondria of mammalian cells in vivo. Selective ablation of the two IRPs, specifically in hepatocytes causes mitochondriopathy with mitochondrial iron deficiency and dysfunction associated with alterations of the ISC biosynthetic pathway, including reduced activity of complexes I, II, and III of the electron transport chain and numerous enzymes of the tricarboxylic acid (TCA) cycle. In knockout mice, this leads to liver failure and death between 8 and 12 days after birth [41].

Interestingly, although *Irp1* knockout mice were initially found to slightly misregulate iron homeostasis in only two tissues (brown fat and kidneys) [42], they have recently been diagnosed with fasting hypoglycemia and shown to contain more erythroid progenitor cells in their spleen than wild-type mice [43]. Although is not yet known whether these defects are caused by the lack of IRP1 IRE-binding activity or its aconitase activity [43], it is tempting to speculate that IRP1 can play a role in earlier developmental stages.

Some intriguing results have been obtained using Cre/Lox technology to generate viable mice lacking the two IRPs in the intestine [44]. Cells lacking both IRPs have decreased DMT1 and TfR1 levels and increases in both Ft subunits and Fpn, and consequently misregulate iron import, export, and storage. As anticipated, these mice develop intestinal malabsorption and dehydration, and die within 4 weeks of birth [44]. Therefore, to study the functioning of the IRP/IRE system in the intestine of adult mice, Ferring-Appel et al*.* [45] used a cre-deletor mouse strain with intestinal-specific expression of a tamoxifen-inducible Cre recombinase to create mice with a ligand-inducible IRP knockout in a single tissue, the intestine. Despite the lack of IRPs, erythropoietin (EPO) stimulation of the knockout mice still increased Fpn and DMT1 levels and decreased L-Ft expression in the enterocytes. This finding indicates that, although IRPs are indispensable for the control of basal expression of iron transporters in the duodenum, they are not responsible for their regulation in response to increased body iron requirement, e.g., during erythropoiesis [45].

### Systemic heme turnover as an integral part of body iron homeostasis

Systemic heme turnover emerges as a crucial element in iron metabolism. The identification of a physiological role for a number of recently identified transmembrane proteins implicated in the intracellular transport of heme, as well as its export to the extracellular environment (for review, see 46), is of the utmost importance for a thorough understanding of systemic iron homeostasis.

Iron fulfills its biological function in the form of iron–sulfur clusters and heme, the most crucial and versatile cofactors found in all life forms. Heme, a ferrous iron protoporphyin IX complex, is an essential molecule in aerobic organisms. It is employed as a prosthetic group in a number of diverse proteins involved in important physiological processes, such as oxygen transport and storage, electron transfer, signal transduction, and microRNA processing [47, 48]. Heme is synthesized in all cells through a series of highly conserved reactions beginning with the condensation of glycine and succinyl-CoA by ALA synthase (ALAS) to form 5-aminolevulinic acid, and continuing through successive enzymatic steps that end with the insertion of iron into the porphyrin ring catalyzed by ferrochelatase.

Similarly to elemental iron, heme is frequently referred to as a two-faced, essential but potentially hazardous, molecule. The toxicity of free heme derives from its lipophilic and hydrophobic properties, and from the iron atom contained within the porphyrin ring. Heme readily enters cellular membranes, catalyzing the oxidation of low-density lipoproteins to cytotoxic oxidized products, with its iron prone to participate in the production of reactive oxygen species (ROS) via the Fenton reaction. To avoid the accumulation of harmful levels of cellular free heme (>1 μm), its concentration is held at the lowest level sufficient to maintain its regulatory functions (estimated at 0.1 μM; i.e., a concentration slightly lower than that of the labile iron pool) [49]. The cellular heme content is mainly regulated via the heme oxygenase (HO) enzyme system [46, 50, 51]. HO catalyzes the rate-limiting step in the heme degradation pathway, resulting in the formation of iron, carbon monoxide, and biliverdin. Two isoforms of the HO enzyme have been identified in mammals: inducible HO-1, and constitutively expressed HO-2. HO-1 is found in most tissues and appears to be largely responsible for heme catabolism following erythrophagocytosis of senescent red blood cells (RBCs) by tissue macrophages [50, 51]. Conversely, HO-2 has a narrow tissue distribution, exhibiting high expression levels in the brain and testes. Recent evidence suggests that cellular heme content may be down-regulated by the plasma membrane heme exporter FLVCR, which was initially identified as the feline leukemia virus sub-group C receptor. The role of FLVCR in efficient heme export has been proven in erythroid colony-forming unit cells [52, 53] and macrophages that ingest senescent RBCs [53]. Considering that the majority of iron (about 70 %) in the body is present in the form of heme-containing proteins (hemoglobin, myoglobin, and cytochromes), it is not surprising that defects in heme synthesis and/or degradation result in perturbations of systemic iron homeostasis, such as iron overload observed in erythropoietic porphyria [54], or tissue iron redistribution associated with HO-1 deficiency [55], respectively. It seems that the contribution of heme to the overall trafficking of iron in the body extends beyond the main points of contact between the heme and iron metabolisms, i.e., recycling of hemoglobin-derived heme iron from senescent erythrocytes and heme synthesis occurring in the erythroid cells of the bone marrow. There is growing evidence that mammals are equipped with a complex molecular machinery responsible for heme turnover, which functions in a similar way to the system responsible for the turnover of elemental iron. In the plasma, heme is transported by the high-affinity heme-binding protein, hemopexin, synthesized mainly in the liver. Hemopexin–heme complexes are removed from the circulation by a process mediated by the scavenger receptor LDL receptor-related protein (LRP1/CD91) [56, 57]. This receptor is expressed in most cell types, indicating that heme may be taken up by multiple tissues in the body. It is noteworthy that once delivered into the cells—hepatocytes, in particular—heme is released into the cytoplasm where it can be used for the reconstitution of newly synthesized hemoproteins or is degraded by HO [57]. Hemopexin is mainly considered a plasma protein that plays a well-established biological role in sequestering heme released into the plasma from hemoglobin as a result of intravascular hemolysis. However, a recent study clearly showed that hemopexin preferentially increases the efficiency of heme export via FLVCR, and thus plays a physiological role in heme iron recycling, which may be of importance for systemic iron homeostasis [58].

Another process, which inserts heme iron into the systemic iron balance is heme absorption. Heme iron serves as an efficient and abundant source of dietary iron in mammals. It is well known that about two-thirds of European dietary iron intake is derived from heme, but the mechanism(s) by which enterocytes take up heme and catabolize it to utilize the iron is still poorly understood. Recent studies on anemic piglets [59] and adolescent girls [60] clearly showed that the bioavailability of heme iron given as a dietary supplement was greater compared to ferrous sulfate and efficiently improved their hematological status. Similarly, the advantage of heme over elemental iron supplementation has also been demonstrated in pregnant woman [61]. Moreover, iron utilization from heme by pregnant women has been shown to be relatively insensitive to hepcidin concentrations or iron stores compared with ferrous sulfate [61]. The high bioavailability of dietary heme iron strongly implies the existence of a specific pathway for heme iron absorption involving heme carrier molecules. However, the results of studies aimed at identifying heme transporters expressed in the apical membrane of duodenal epithelial cells are controversial [62, 63]. Interestingly, intriguing recent results show that, during human pregnancy, the fetus preferentially uses iron absorbed by the mother in the form of heme compared to iron ingested as ferrous sulfate [64]. The authors hypothesized that this may be a consequence of greater intestinal heme Fe uptake in the mother, which may involve the transport of intact heme through absorptive enterocytes into the circulation and then its transfer across the placenta to the fetus. Accordingly, the expression of FLVCR in term placenta obtained from pregnant adolescents has been found to be inversely associated with maternal iron status and placental iron concentration, suggesting the functional role of this protein in placental heme transport [65].

## Molecular basis of fetal iron metabolism

As in the case of adults, the main insights into the molecular mechanisms of iron metabolism in the fetus have come from the study of various mouse models with disrupted iron metabolism genes. It is not surprising that a deficiency of genes encoding proteins critically important for the regulation of cellular iron storage and transport, such as H-ferritin (H-Ft) [66], transferrin receptor 1 (TfR1) [67] and ferroportin (Fpn) [68], causes lethality at an early stage of embryonic development. As mentioned above, ferritin is a cytosolic protein ubiquitously distributed among living species. The H-ferritin chain possesses ferroxidase activity and readily interacts with Fe(II) to induce its oxidation and deposition inside a large protein shell in a non-toxic and bioavailable form. Mouse embryos homozygous for a null allele of H-Ft die between days 3.5 and 9.5 of development. A possible reason for this lethality in the absence of H subunits is that iron entering embryo cells cannot be internalized and sequestered inside the large cavity of ferritin molecules, so is available to participate in the Fenton reaction, which leads to the exacerbation of oxidative stress [66]. The opposite scenario with regard to ferritin expression has been observed in mouse embryos with a double knockout of the *Irp1* and *Irp2* genes, the two repressors of ferritin mRNA translation. The lethality at the pre-implantation stage (6.5 days) observed in blastocytes lacking two functional IRPs has been attributed to ferritin overexpression, increased iron sequestration, and concomitant functional iron deficiency [40]. It was hypothesized that the low availability of iron in *Irp1* and *Irp2* null blastocytes may be further decreased by reduced uptake of extra-embryonic iron due to the degradation of TfR1 transcripts in the absence of IRPs [40]. It is noteworthy that, in the absence of either IRP1 [42] or IRP2 [37, 42], the posttranscriptional regulation of iron-related genes seems to proceed normally at all stages of prenatal development, presumably due to the functional redundancy of the IRPs.

TfR1 is a second pillar of cellular iron homeostasis, and its indispensability for the progression of fetal development is demonstrated by the death of TfR1 knockout embryos between days 8.5 and 12.5 of development [58]. Studies on the localization of TfR1 in early embryonic life after the implantation of the blastocyst showed expression in the embryonic ectoderm and in syncytiotrophoblasts, which are derived from trophoblast cells and take part in the formation of the placenta, the site of materno–fetal iron transfer [67]. The localization of TfR1 suggests that TfR1-mediated uptake of Fe-transferrin complex is crucial for post-implantation mouse development and beyond.

The major cellular iron exporter, ferroportin (encoded by the *Slc40a1* gene), is also essential for the development of the mouse embryo [68, 69]. Global targeted inactivation of the murine *Slc40a1* results in embryonic lethality before the establishment of the placenta, which occurs by E9.0–E9.5 [68]. Immunohistochemical localization of ferroportin in wild-type embryos demonstrated that this protein is strongly expressed on the basolateral surface of polarized epithelial cells, which constitute the extraembryonic visceral endoderm. This structure is responsible for materno–embryonic delivery of nutrients, including iron, prior to placenta formation. Interestingly, failure of embryonic development was not observed following selective inactivation of the *Slc40a1* gene in the embryo proper. Taken together, these data clearly indicate that ferroportin functions as a major protein transporting iron from the mother to the embryo/fetus.

Another gene that is indispensable for embryo development is *Flvcr*. Prenatal death of *Flvcr* ^−*/*−^ mouse embryos occurs during one of two embryonic development stages: at or before E7.5, or between E14.5 and E16.5 [53]. Death during the latter stage is caused by the failure of fetal erythropoiesis in the liver, which is consistent with the functioning of FLVCR as a heme exporter playing a crucial role in protecting erythroid cells from heme toxicity. In normally developing embryos, FLVCR is expressed in the yolk sac, the ectoplacental cone, and the placenta [53].

HO-1 (encoded by the *Hmox1* gene) is one of the molecules that seem to be important, although not indispensable, for promoting placenta function and successful fetal development [70]. Mating of *Hmox1* ^+*/*−^ mice results in the production of *Hmox1* ^−*/*−^ progeny at a frequency below the expected Mendelian distribution (6–20 %, depending on the genetic background of the mice [71]), which indicates non-negligible prenatal lethality. It is also noteworthy that the in vitro fertilization rate of *Hmox1* ^−*/*−^ oocytes with wild-type sperm is very low (19.78 %)*.* Moreover, *Hmox1* ^−/−^ females fail to become pregnant when interbred with *Hmox1* ^−/−^ males [72]. A role for HO-1 in embryo implantation has been suggested by some studies. Indeed, significant HO-1 expression is detected in the extra-embryonic tissues during early fetal development, particularly in the ectoplacental cone at E6.5 and in the placenta of E13.5–14.5 embryos [73]. HO-1 expression then shows a marked decline in the placenta of older embryos until the end of pregnancy [73]. It has been noted that HO-1 expression parallels that of FLVCR in extra-embryonic tissues during early development, which suggests that they may perform a coordinated function to lower the heme level and thus to prevent its toxicity [53]. However, it should be remembered that, apart from its activity in reducing heme toxicity, HO-1 displays anti-oxidant, anti-inflammatory, and cytoprotective functions that may also be beneficial to the developing embryo.

Divalent metal transporter 1 (DMT1, encoded by the *Slc11a2* gene) is a transmembrane glycoprotein, which mediates the proton-coupled transport of a variety of divalent metal ions, among which ferrous ions appear to be its most important physiological substrate. It is expressed at the apical membrane of duodenal enterocytes and in recycling endosomes of most cell types, especially in erythroid precursors, where it mediates the transfer of iron internalized by transferrin from the endosomes to the cytoplasm [74]. Although *Slc11a2* ^−/−^ mice are born anemic (microcytic hypochromic anemia) and do not survive beyond 7 days, the iron content in most of their tissues appears normal or even higher than in wild-type mice [75]. This means that the function of DMT1 is dispensable for materno–fetal iron transfer across the placenta, but is crucial for erythroid iron utilization.

Surprisingly, a number of mouse mutants with disruption of genes important for adult iron homeostasis, such as HFE [76], hemojuvelin [77], haptoglobin [78], hemopexin [79], hepcidin [9, 80], and ceruloplasmin [81], exhibit neither overt fetal abnormalities nor prenatal lethality, and produce fertile homozygous offspring in the expected ratio.

## Molecular control of non-heme iron transport across the placenta

The growth of the fetus requires constant delivery of iron, in amounts which markedly increase towards the end of pregnancy (about 5 mg of iron per day are required at term gestation in humans). It has been suggested that, from the start of mouse embryonic development up to the 3.5-day blastocyst stage, iron is taken from the maternal ferritin present in the oocyte [66]. Subsequent acquisition of iron by the embryo and fetus relies on materno–embryonic and materno–fetal transfer of this microelement across the extraembryonic visceral endoderm and the placenta, respectively. Despite recent advances, the materno–fetal iron transfer at the placenta level and its regulation remain the most poorly understood aspects of mammalian iron metabolism (for review, see [82]).

**Fig. 2:**
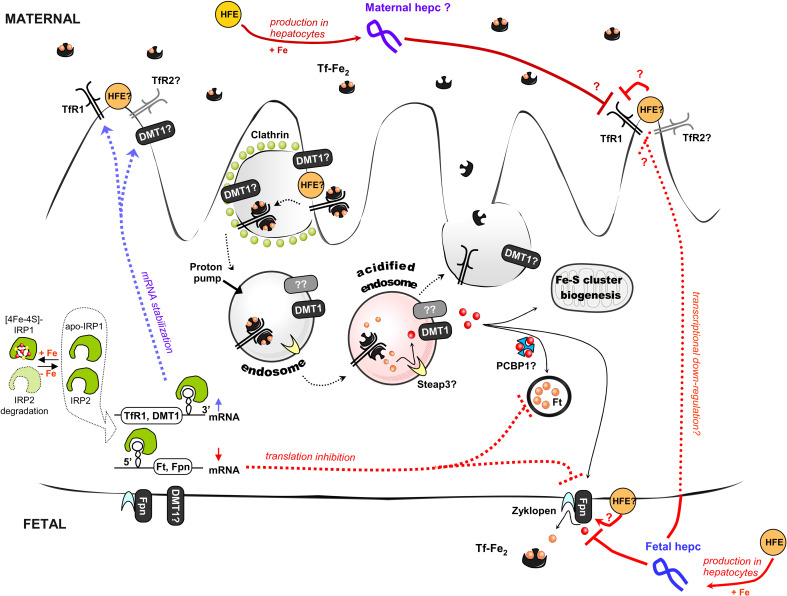
Iron transport across the placenta (syncytiotrophoblasts). Diferric transferrin (Tf-Fe_2_) from the maternal blood binds to Transferrin Receptor 1 (TfR1) and is taken up by syncytiotrophoblasts via clathrin-mediated endocytosis. Inside the cell, specialized endosomes are formed and subsequently acidified by a proton pump. At pH 5.5, iron is released from transferrin molecules, while the transferrin itself remains bound to TfR1. Subsequently the Tf, in complex with TfR1, is recycled back to the cell surface, where, at the higher pH, its affinity for the receptor is reduced and it disassociates. Iron released from transferrin inside the acidified endosome is reduced to the ferrous state (Fe^2+^) by an oxidoreductase (potentially Steap3 in syncytiotrophoblasts) and is then transported to the cytoplasm via DMT1 or another as yet unknown transporter (*question mark* in the figure). Once in the cytoplasm, iron can be stored in ferritin (Ft), used for iron–sulfur cluster biogenesis and heme synthesis, or exported to the fetal circulation by ferroportin (Fpn), which, in syncytiotrophoblasts, acts in cooperation with zyklopen, a copper-dependent ferroxidase. The mRNA transcript encoding the second transferrin receptor (TfR2) has also been detected in the placenta, but the role of the TfR2 protein in iron import by syncytiotrophoblasts is elusive. Iron transport through the placenta is regulated at several levels. In response to changes in the intracellular iron pool, the iron regulatory proteins (IRPs) can regulate the expression of target genes (TfR1, DMT1, Ft, and Fpn) at the post-transcriptional level. In the absence of iron, both IRPs bind to specific sequences, called iron responsive elements (IREs), located within the untranslated regions (UTRs) of target mRNAs. Binding to the 3′UTR IRE increases mRNA stability (e.g., for TfR1 or DMT1), whereas binding to the 5′UTR IRE blocks its translation (which is the case for Ft and Fpn). When iron is abundant, IRP1 assembles an iron–sulfur cluster and IRP2 is degraded by a FBXL5-dependent pathway. Both maternal and fetal hepcidin levels seems to regulate the rate of iron trafficking through the placenta. Fetal hepcidin most probably acts by binding to Fpn located in the basolateral membrane of syncytiotrophoblasts, thus promoting its internalization and subsequent degradation. Moreover, fetal hepcidin was also proposed to down-regulate TfR1 expression at the apical membrane of syncytiotrophoblasts by an unidentified transcriptional mechanism. In addition, hereditary hemochromatosis protein (HFE), a known regulator of hepcidin expression in hepatocytes, was recently shown to be an important player in the modulation of iron transfer across the placenta. Several mechanisms have been proposed for this regulation, depending on the source of the protein, i.e., maternal or fetal. Since syncytiotrophoblasts are genetically fetal in origin, and HFE protein was found to be expressed and interact with TfR1 at the syncytiotrophoblast apical plasma membrane, it was proposed that, in placenta, fetal HFE can compete with transferrin for the binding site on TfR1 and thus negatively regulate maternal–fetal iron transfer. In contrast, HFE was proposed to localize at the basal membrane of human syncytiotrophoblasts and furthermore, colocalize with ferroportin, although its potential role at this location has not been elucidated. HFE, as an important component of a larger iron-sensing complex at the plasma membrane of hepatocytes, can govern the regulation of fetal and maternal hepcidin expression. However, this axis seems to be important only for fetal hepcidin expression, since *Hfe* knockout pups show a close relationship between very low expression of hepatic hepcidin and high levels of placental ferroportin. Interestingly, although maternal HFE (*yellow*) seems to regulate the expression of TfR1, DMT1, and Fpn in the placenta of dams fed a high iron diet, its inactivation neither changes the expression of TfR1, DMT1, and Fpn in the placenta in animals fed a low iron diet, nor does it modulate maternal hepcidin expression

The placenta is a highly specialized transitory yet indispensable structure, which primarily promotes the exchange of nutrients and gases between maternal and fetal compartments, a process that is essential for fetal growth and survival. It is composed of both zygote-derived and maternal cells, and attaches the conceptus to the uterus. The structure of this organ varies remarkably across species [83]. The human hemochorial placenta is composed of a single layer of fused polarized cells called syncytiotrophoblasts, which are directly connected with the maternal vascular system. These cells originate from an underlying layer of cells called the villous cytotrophoblasts. The fetal capillary endothelium lies close to the basal side of the syncytiotrophoblasts [83]. The first step in iron transport across the placenta is traversal of the microvillous apical plasma membrane of the syncytiotrophoblasts (Fig. 2). Once in the cytoplasm, iron exits from the syncytiotrophoblasts via the fetal-facing basal plasma membrane [82]. The expression and activity of iron transporters within these two plasma membranes provide the basis for vectorial transport towards the fetus. Maternal iron is then transferred across the placenta via a specialized molecular machinery. Iron-loaded (diferric) transferrin (Tf-Fe_2_) binds to the Tf receptor 1 (TfR1), which is highly and predominantly expressed on the apical (maternal) membrane of the syncytiotrophoblasts [84, 85], and enters the cell by clathrin-mediated endocytosis. Inside the cell, the TfR1–Tf-Fe_2_ complex is trafficked to early endosomes, delivers iron by a process that involves endosomal acidification, and is subsequently directed to recycling endosomes and transported back to the cell surface. Although it is largely accepted that the reduction of released ferric iron is an essential step in the transferrin cycle (the endosomal ferrireductase required for efficient Tf-dependent iron uptake in erythroid cells has recently been identified [86]), it is not yet known how the conversion of iron to its ferrous form is achieved in the syncytiotrophoblasts. As mentioned above, ferrous iron is transported out of the endosome into the cytoplasm by DMT1 in most cell types. DMT1 is also expressed in the placenta, where it has been implicated in materno–fetal iron transfer [84, 85, 87, 88]. In human placenta, DMT1 is found in the cytoplasm [87] and at the fetal (basal) membrane of the syncytiotrophoblasts [84, 87]. Only a small overlap in the localization of TfR1 and DMT1 has been found in human syncytiotrophoblasts [84], and, accordingly, it was proposed that DMT1 transfers iron out of the endosome and across the basal membrane to the fetus. However, studies with knockout mice clearly indicate that a DMT1-independent iron uptake pathway must also be active in the placenta [75].

There is increasing evidence that ferroportin, the sole iron exporter, which is highly expressed on the basolateral membrane of absorptive enterocytes and the plasma membrane of macrophages, is also present in syncytiotrophoblasts [84, 85]. In the human placenta, Fpn occupies the basal membrane of the syncytiotrophoblast [84], which is consistent with its role in iron export to the extracellular environment, i.e. from the syncytiotrophoblasts into the fetal circulation. It is not yet known exactly how iron passes across the barrier of the fetal vascular endothelium to enter the fetal circulation from the syncytiotrophoblasts. However, as underlined by McArdle [82], this step may be crucial in the passage of iron from the mother to the fetus. Importantly, Fpn is not expressed on fetal blood vessels in humans [84]. In enterocytes and macrophages, ferroportin is assisted by a ferroxidase activity of hephaestin and ceruloplasmin, respectively, to deliver iron in the ferric form to fetal plasma transferrin. In syncytiotrophoblasts, Fpn seems to cooperate with another copper-dependent ferroxidase, zyklopen, recently identified in mouse placenta [89].

During fetal development, the iron requirements of the fetus must be matched by the transport of maternal iron across the placenta. The rat model of the regulation of this complex process clearly indicates that the maintenance of adequate iron levels in fetal tissues (including hepatic iron stores) is the highest priority in the hierarchy of iron delivery during pregnancy [82, 90]. The need for iron to support the hematological status of the mother is next in this hierarchy, followed by the maintenance of iron stores in the maternal liver. In the light of recent advances, the regulation of iron transfer across the placenta emerges as a subtle interplay governed by both mother and fetus [82]. It is highly likely that TfR1 and ferroportin, two iron transporters expressed, respectively, on the apical and basal plasma membranes of the syncytiotrophoblasts, are the main molecular targets of regulation. However, the amount of iron transferred across the basal membrane by ferroportin may also be modulated, at least temporarily, by cytosolic ferritin due to its high potential to store iron. Ferritin is not expressed at a high level in human syncytiotrophoblasts [84], suggesting that most iron entering these cells is not stored, but is immediately transported to the fetus.

Possible mechanisms modulating the expression of genes implicated in placental iron transfer include transcriptional regulation, post-transcriptional regulation through the IRP/IRE system, and downstream regulation by hepcidin and by the hereditary hemochromatosis protein (HFE).

Placental TfR1 is a gatekeeper at the syncytiotrophoblast apical membrane that controls the initial step in iron uptake from the mother to the fetus. Its expression at both the mRNA and protein levels is up-regulated by iron deficiency caused by maternal dietary iron limitation in pregnant rats [90, 91]. In contrast, parenteral supplementation of pregnant mice with iron [92] and exposure to an iron-adequate diet [91] lead to decreased TfR1 mRNA levels. This bi-directional regulation of TfR1 probably results from the differential iron status in the placenta, which stabilizes the TfR1 mRNA (iron deficiency) or promotes its degradation (iron replenishment) through the IRP/IRE intracellular regulatory system. Accordingly, in iron-deficient placentae from diabetic mothers, increased IRP1 IRE-binding activity was found to closely correlate with an increased TfR1 mRNA concentration [93]. In human placentae at 24–40 weeks of gestation, the activities of IRP1 and IRP2 are regulated in a predictable manner by the placental iron status [94]. The involvement of IRPs in the regulation of DMT1 mRNA expression is supported by the fact that the IRE-DMT1, but not non-IRE-DMT1, mRNA isoform levels, were increased in rat placenta in response to iron deficiency [90]. The IRE-regulated form of DMT1 (mRNA contains IRE in its 3′UTR) is predominantly expressed in human and rat placentae [88, 90].

The unexpected regulation of placental TfR1 has been reported in transgenic mice overexpressing hepcidin [92]. Transgenic embryos overexpressing hepcidin suffer from severe iron deficiency anemia (IDA) and die at around the time of birth [9]. Placental TfR1 was found to be strongly down-regulated in these embryos, and it was consequently hypothesized that their critically poor iron status is the result of reduced iron uptake on the maternal side. Importantly, it was demonstrated that the hepcidin-mediated decrease in TfR1 expression is IRP-independent, and this suggests that hepcidin may indirectly influence the expression of the *TfR1* gene through transcriptional down-regulation. At the time these results were published (August 2004), the molecular mechanism of hepcidin action had not yet been elucidated; the inhibition of cellular iron efflux by binding of hepcidin to ferroportin was reported in December 2004 [95]. It now seems obvious to propose that fetal hepcidin-mediated down-regulation of iron efflux from the placenta proceeds through its interaction with ferroportin at the basolateral membrane of syncytiotrophoblasts. However, this mechanism has yet to be verified in transgenic embryos overexpressing hepcidin, which would seem to be the most suitable experimental model for this purpose. A perhaps more interesting observation is that in mice with targeted disruption of the *Hamp* gene, severe iron accumulation is not manifested prenataly, but appears only after birth, leading gradually to hemochromatosis at the age of a few months [9, 80]. This implies that in the absence of fetal hepcidin, the uncontrolled efflux of iron to the fetus is counteracted by decreased iron uptake from the mother. It is uncertain to what extent fetal hepcidin, originating from placenta, participates in the regulation of iron transport from the fetus to the mother. In humans, immunohistochemical localization of hepcidin in the first-trimester placenta revealed its presence in the syncytiotrophoblasts as well as in mesothelial and endodermal layers of the secondary yolk sac at 10 weeks [96]. Authors suggested a key regulatory role for this protein in iron transfer to the first-trimester fetus. On the other hand, studies on pregnant rat females assign no role for placental hepcidin in the modulation of iron uptake from the maternal blood. [90].

It may be speculated that the fetal hepcidin–placental ferroportin axis represents an important element in the fetus-dependent control of iron transport through the placenta. The expression of hepcidin mRNA has been shown to be decreased in the livers of fetuses from dams fed with iron-deficient diets compared with those on an iron-supplemented diet [85]. This suggests that low fetal hepcidin expression could contribute to the higher ferroportin expression at the basal membrane of syncytiotrophoblasts and, in consequence, increase iron efflux from these cells. Recent data show that the fetal hepcidin–placental ferroportin regulatory axis does indeed function at higher dietary iron levels [91]. It should be remembered that, apart from the downstream regulation of ferroportin by hepcidin, other mechanisms, such as transcriptional regulation by heme, as well as post-transcriptional regulation through the IRP/IRE system [97], may contribute to the final ferroportin expression profile. Indeed, in nearly full-term human placenta, increased expression of both Fpn and ferritin are correlated with decreased IRP1 IRE-binding activity according to the pattern of regulation of mRNAs containing IRE regulatory sequences in their 5′UTRs [94].

Several lines of evidence indicate that HFE is a modulator of iron transfer across the placenta. The hereditary hemochromatosis protein, HFE, which is responsible for type 1 hemochromatosis, was identified over 15 years ago [98]. Although this protein has been extensively studied, its function is only just being elucidated. HFE is a positive modulator of *Hamp* transcription, which, when defective, leads to hemochromatosis (HH) in humans and a HH-like phenotype in knockout animal models. The most common mutation in HFE is a single nucleotide change resulting in a C to Y substitution at amino acid 282. Recent studies have clarified the crucial role of HFE as a hepatocyte iron sensor and upstream regulator of hepcidin [99, 100], and several mechanisms by which this protein may regulate iron metabolism have been proposed. It may compete with transferrin for binding to TfR1, thus lowering iron uptake into cells [101, 102]. Alternatively, there is more recent evidence supporting a role for HFE as an important component of a larger iron-sensing complex that involves interactions with diferric transferrin and TfR1 and TfR2 at the plasma membrane of hepatocytes [103, 104]. In this scenario, defective HFE prevents the formation of a functional iron sensor and signal transduction complex leading to dysregulated hepcidin expression as observed in human hereditary hemochromatosis [105, 106] and mouse models of this disease [76].

HFE protein is expressed in human placenta at the apical plasma membrane of the syncytiotrophoblasts [107], where it interacts with TfR1 [108]. These findings raise the possibility that, as mentioned above in the case of hepatocytes, HFE competes with transferrin for the same binding site on TfR1 and thus negatively regulates maternal–fetal iron transfer. Contrary to these findings, another study showed that HFE is present at the basal membrane of human syncytiotrophoblasts and colocalizes with ferroportin, but not TfR1 [84]. Accordingly, HFE expression in macrophage cell lines and in HT29 cells (an intestinal cell line) inhibits iron efflux from these cells. The distinct and non-overlapping patterns of localization of HFE and TfR1 in syncytiotrophoblasts imply that any association between these proteins is minimal. The findings of a very recent study, in which *Hfe* wild-type, knockout, and heterozygote dams were mated with heterozygote males to produce pups of all genotypes, provide some insight into the role of fetal and maternal HFE in modulating the passage of iron across the placenta [91]. *Hfe* knockout pups showed a close relationship between very low expression of hepatic hepcidin, a high level of placental ferroportin at both the mRNA and protein levels, and a high iron content in the fetal liver [91]. This relationship is consistent with the functional pattern of the hepcidin–ferroportin regulatory axis. The effect of inactivation of the maternal *Hfe* gene on the iron loading of the *Hfe* heterozygote fetus was only observed when the mothers were fed a high iron diet (50 ppm). In this case, placental expression of all examined iron transporters (TfR1, DMT1, and Fpn) in *Hfe* heterozygote pups was higher than in those derived from Hfe wild-type dams. It seems that the fetal–maternal HFE-dependent regulation described by Belasaria et al. [91] may be relevant in subjects with different allelic variants of HFE exhibiting different levels of iron overload [109].

## Iron metabolism in early postnatal life

In most mammals, systemic iron homeostasis is essentially a closed system. This means that iron recycling by tissue macrophages, that phagocytose senescent erythrocytes and degrade hemoglobin and heme, provides sufficient iron to meet the needs of erythroid precursors, the primary iron consumers in the body. Under physiological conditions, daily iron losses are negligible and do not involve regulated pathways for iron excretion through the liver in bile and/or through the kidney in urine. This imposes a strict control on iron uptake to prevent iron excess and toxicity, which is mainly achieved by minimizing intestinal absorption. In contrast, in the neonatal period, intestinal iron absorption of dietary (exogenous) iron is an important way to meet the needs of the rapidly growing organism, particularly the increase in blood volume and the number of RBCs. The function of the molecular machinery involved in intestinal iron transport and its regulation during early life have been recently reviewed by Collard [110]. The article concludes with a statement that our understanding of these processes in the neonatal period of mammalian development is poor. The few studies that have been performed strongly indicate that the expression of the main iron transporters in the duodenum is very low during the neonatal period. In mice, the DMT1 protein is barely detectable at postnatal days 0 and 5, but by day 10, this transporter is already predominantly localized in the apical membrane of the maturing intestine [111]. Similarly, in newborn piglets, a strong DMT1 signal is detected only on day 7 after birth in the villi at the apical site of enterocytes, corresponding to the brush border [112]. Fpn is found exclusively at the basolateral membrane of porcine absorptive enterocytes and, similarly to DMT1, its expression is increased on day 7 after birth [112]. Developmental regulation of both intestinal iron transporters has been also studied in rats [113, 114]. The expression of both rat genes increases dramatically only on day 40 after birth [114]. Interestingly, these developmental changes in the expression of DMT1 and Fpn were found to be far greater than those induced by dietary iron supplementation [114]. Moreover, the response of intestinal DMT1 and Fpn expression to dietary iron also seems to be developmentally dependent. In 10-day-old rats, the expression of duodenal DMT1 and Fpn is not regulated by an increased iron content in the diet. The expected regulation of DMT1 and Fpn expression in response to both an iron-supplemented and an iron-deficient diet are observed only on day 20 after birth [113, 114]. Furthermore, the unexpected up-regulation of duodenal Fpn and DMT1 in piglets receiving intramuscular iron injections on day 4 after birth also indicates that the regulation of duodenal iron absorption during early life might differ from that during adulthood [112]. Taken together, these findings underline the need for careful consideration before giving iron supplements to neonates and infants, in order to avoid the potential toxic effects of iron.

Since the developmental maturation of the DMT1-dependent pathway of iron absorption occurs a few days after birth, it has been suggested that there may be alternative sources of iron for newborns [111]. Lactoferrin, which is a major iron-binding glycoprotein abundantly present in human milk, was postulated to be involved in intestinal iron absorption in breast-fed infants and in suckling newborn animals. This protein also represents a promising candidate for an alternative iron source in the absence of a functional DMT1 pathway. The identification of a specific receptor for lactoferrin (LfR) in the small intestine of newborn infants [115] and suckling piglets [116] is evidence that the Lf-LfR pathway plays a role in iron absorption during early life. However, a comparison of the iron status of suckling progeny from mothers with a disrupted *Lf* gene and those from wild-type mothers showed that lactoferrin is not essential for iron absorption during the early postnatal period and does not play a major role in the regulation of this process [117]. This conclusion is supported by the observation that hemoglobin levels in 10-day-old suckling mouse neonates receiving milk from transgenic mothers overproducing lactoferrin or from mothers with a normal Lf content in their milk are not significantly different [118]. Earlier studies on the role of lactoferrin in iron absorption in 2- to 10-month-old infants fed breast milk and the same milk from which lactoferrin had been removed do not support a direct role for Lf in the enhancement of iron absorption from human milk [119].

Since the molecular potential of iron uptake in neonates is greatly reduced and the ability to adjust iron absorption to dietary supply is not fully developed, it appears that hepatic iron stores represent the primary source of this microelement to cope with the metabolic demands of the organism. In other words, the initial iron stores established through materno–embryonic and materno–fetal transfer determine the iron status of the newborn. As mentioned above, the amount of iron transferred from mother to fetus increases during pregnancy. Thus, in humans, the neonatal iron status is primarily a function of third-trimester maternal–placental–fetal iron transport. Another important factor that influences the iron reservoir of human newborns is the amount of blood transferred from the placenta before the umbilical cord is clamped. A newborn’s blood volume can be increased by up to 32 %, and thus an extra 30–50 mg of iron can be transferred if the clamping is performed with a delay [120]. Importantly, the replacement of “fetal-type” hemoglobin with the “adult-type” occurs in the perinatal period (fetal Hb production decreases and adult Hb production increases, reaching approximately 98 % of total Hb 20–30 weeks postnatally) and is associated with an increase in the phagocytic activity of tissue macrophages. The use of iron stored in hepatocytes, as well as iron recovered after erythrophagocytosis by Kupffer cells, implies an extremely efficient mechanism of iron release from these cells and points to a critical role for both hepatic HO-1 and Fpn in iron homeostasis in neonates. However, this speculation has not yet been confirmed.

Despite the well-established pivotal role of hepcidin in the control of iron absorption and recycling in adults, the regulation of hepcidin expression and release during the neonatal period is poorly understood. It is not even clear whether the main mechanism underlying the maintenance of the systemic iron balance relies on hepcidin-dependent regulation. Hepcidin expression is modulated by different factors, which act as positive or negative regulators. At least four regulatory pathways control hepcidin synthesis through numerous signaling molecules: the iron stores pathway, the erythroid pathway, the hypoxia pathway, and inflammation-mediated regulation [21–23]. All these signaling pathways appear to be active in newborn piglets, although the exact nature of the crosstalk between them is currently unclear. Interestingly, high levels of Hepc mRNA have been observed in rats [121], mice [8], and pigs [112] in the perinatal period. The high Hepc expression is particularly puzzling in newborn piglets considering their low hepatic iron content. It might be partially explained by the fact that the birth process initiates an acute phase response in a healthy fetus/newborn, which is characterized by increased circulating levels of interleukin-6 [122], a well-known inflammatory cytokine that is responsible for the induction of Hepc during inflammation [123]. However, it is important to stress that, in most animal studies, Hepc expression has been examined at the mRNA level, and the correlation with circulating levels of bioactive Hepc (a 25 amino acid peptide) remains largely unknown. Several serum Hepc assays have been developed, mostly for humans [124]. Available data from the quantification of plasma hepcidin in the neonatal period [125–127] are inconclusive with regard to its role in the iron metabolism of neonates. The results of a large study (191 human newborns) aimed at determining hepcidin concentration in cord blood of newborns at term show that the concentration of this peptide is generally appropriate for the fetal iron status and decreases with decreasing fetal iron stores [125]. Other studies show no correlations between serum hepcidin levels and serum iron parameters in human newborns [126, 127]. Further information on hepcidin regulation and function in the neonatal period is vital in order to increase our understanding of the early developmental changes in iron metabolism in mammals.

## Concluding remarks and proposals

The aim of this review was to compile and analyze the limited information available on the role of genes involved in iron metabolism during the initial stages of ontogenic mammalian development. Based on the results from knockout mouse models, a panel of relevant genes that are indispensable for growth of the fetus was identified. We have also outlined the key elements involved in the complex and still poorly understood regulation of materno–embryonic/fetal iron transfer: a process that is under the control of molecular mechanisms originating from two “control centers”, i.e., the fetus and the mother. Finally, we provide evidence that, in newborns and infants, the molecular machinery responsible for iron absorption is not fully developed, does not respond to iron-dependent regulation, and thus has a reduced ability for exogenous iron uptake.

Obviously, apart from the molecular capacities of the organism, there are multiple etiological factors that positively and negatively determine iron status in the fetus and neonate, which are beyond the scope of this review (for an exhaustive list, see [128]). It is generally considered that healthy term neonates are born with iron stores that are sufficient to support their development during early postnatal life. On the other hand, iron deficiency is most prevalent in the early postnatal period [129] and may have long-lasting (extending beyond infancy) negative effects on brain development and function [130]. To combat this problem, numerous iron supplementation strategies for pregnant/nursing females and neonates/infants have been proposed [129]. However, it is difficult to meet all the criteria of efficient iron supplementation (such as improvement of iron status), while attenuating the risks of iron metabolism misregulation (for example, excessive induction of hepcidin expression), and finally preventing supplemented iron toxicity. We believe that a better understanding of the molecular regulatory network, which functions at the fetus–placenta–mother interface, is required in order to optimize protocols for iron supplementation and therapy in the early neonatal period.

The results of several studies, including our own [112, 131–135], indicate that newborn piglets are a suitable model with which to explore iron metabolism in the neonatal period. First, iron deficiency anemia is the most prevalent deficiency disorder during the early postnatal period in pigs, and frequently develops into a critical illness [136]. It seems that the pig model of IDA accurately reflects this defect observed in pre-term human neonates, as the iron content in their liver is very low [137]. Second, the pig is being increasingly used in biomedical research for studies on human genetic and nutritional diseases, which are not accurately represented by rodent models [138]. Third, the pig genome has been sequenced, and molecular tools are now available for studying iron-related genes in the pig model. On the other hand, it should be kept in mind that several reasons for iron deficiency in newborn piglets, such as the high number of animals in the litter, their rapid growth (particularly the increase in blood volume and the number of red blood cells), and low iron content in sow’s milk [136], do not occur in human newborns and thus inaccurately reproduce human conditions. Finally, it is noteworthy that no cases of iron deficiency have been reported in the offspring of wild boar (*Sus scrofa*), the ancestor of domesticated pigs (*Sus scrofa domestica*), suggesting that the iron metabolism is well balanced in these animals. Wild boars continue to survive and develop in their natural habitat without any iron supplements, whereas the use of parenteral iron supplementation in piglets is a routine practice in the swine industry [137]. Therefore, comparative studies of the iron metabolism in neonates of the domestic pig and wild boar may be highly informative.
